# Deep learning algorithms for predicting renal replacement therapy initiation in CKD patients: a retrospective cohort study

**DOI:** 10.1186/s12882-024-03538-6

**Published:** 2024-03-14

**Authors:** Ka-Chun Leung, Wincy Wing-Sze Ng, Yui-Pong Siu, Anthony Kai-Ching Hau, Hoi-Kan Lee

**Affiliations:** 1 Department of Medicine and Geriatrics, Tuen Mun Hospital, Hong Kong, China; 2 Adult Intensive Care Unit, Queen Mary Hospital, Hong Kong, China

**Keywords:** Artificial intelligence, Chronic renal failure, Data-driven modeling, Predictive analytics, Renal replacement therapy

## Abstract

**Background:**

Chronic kidney disease (CKD) requires accurate prediction of renal replacement therapy (RRT) initiation risk. This study developed deep learning algorithms (DLAs) to predict RRT risk in CKD patients by incorporating medical history and prescriptions in addition to biochemical investigations.

**Methods:**

A multi-centre retrospective cohort study was conducted in three major hospitals in Hong Kong. CKD patients with an eGFR < 30ml/min/1.73m^2^ were included. DLAs of various structures were created and trained using patient data. Using a test set, the DLAs' predictive performance was compared to Kidney Failure Risk Equation (KFRE).

**Results:**

DLAs outperformed KFRE in predicting RRT initiation risk (CNN + LSTM + ANN layers ROC-AUC = 0.90; CNN ROC-AUC = 0.91; 4-variable KFRE: ROC-AUC = 0.84; 8-variable KFRE: ROC-AUC = 0.84). DLAs accurately predicted uncoded renal transplants and patients requiring dialysis after 5 years, demonstrating their ability to capture non-linear relationships.

**Conclusions:**

DLAs provide accurate predictions of RRT risk in CKD patients, surpassing traditional methods like KFRE. Incorporating medical history and prescriptions improves prediction performance. While our findings suggest that DLAs hold promise for improving patient care and resource allocation in CKD management, further prospective observational studies and randomized controlled trials are necessary to fully understand their impact, particularly regarding DLA interpretability, bias minimization, and overfitting reduction. Overall, our research underscores the emerging role of DLAs as potentially valuable tools in advancing the management of CKD and predicting RRT initiation risk.

**Supplementary Information:**

The online version contains supplementary material available at 10.1186/s12882-024-03538-6.

## Background

Chronic kidney disease (CKD) significantly contributes to global mortality, affecting approximately 10% of adults worldwide [1]. In Hong Kong, the burden of chronic illness management on hospital-based internal medicine specialist clinics is notable [2], evidenced by patients in primary care clinics reduced by around 400,000 from 2015 to 2021, while internal medicine specialty clinics increased by around 200,000 patient load in the same period [3]. The referral process in this three-tiered system – from primary care to internal medicine and then to renal clinics – is often guided by primary care and internal medicine physicians using biochemical markers (e.g. serum creatinine, urine protein-to-creatinine ratio) and clinical symptoms (e.g. haematuria, persistent lower limb oedema). However, this referral system may not accurately predict the imminent risk of end-stage renal failure (ESRF), indicating a need for improved prediction methods for progression to ESRF requiring renal replacement therapy (RRT).

Tangri et al. developed a statistical model to predict the risk of initiating RRT over a decade ago [4]. Although its robustness has been validated by multiple localized studies [5, 6], this model did not consider one's previous medical history and ongoing prescriptions, and often faces data availability challenges in typical clinical settings. For instance, the requirement for urine albumin-creatinine ratio in some models may not always be practical. Furthermore, these studies predominantly involved Caucasian populations, raising questions about their global applicability. The recent emergence of new treatments, such as SGLT-2 inhibitors that markedly slow CKD progression, further emphasizes the need for predictive models adaptable to these evolving therapeutic landscapes [7].

Recent computerization and processing power advances have made life easier for clinicians and academic researchers to retrieve and analyze patient records, investigation reports, and prescriptions. The advancement in data science enables the development of Deep Learning Algorithms (DLAs). These DLAs, exemplified by the model of Tomašev et al. for acute kidney injury prediction, can capture intricate multidimensional relationships beyond linear dependencies [8, 9]. Yet, the efficacy of DLAs in predicting RRT risk in CKD patients remains unexplored.

Our study endeavors to develop a DLA that surpasses the Kidney Failure Risk Equation (KFRE), an established risk prediction tool, in predicting RRT initiation [4]. This endeavor is not only a stride in CKD management but also a testament to the evolving landscape of medical data analysis.

## Methods

### Data collection and preprocessing

This multi-centre retrospective cohort study was conducted at Tuen Mun Hospital (TMH), Pok Oi Hospital (POH), and Tin Shui Wai Hospital (TSH), which are major acute hospitals in Hong Kong with a combined total of 3000 beds. The study period was from January 1, 2009, to March 31, 2022. Eligible patients were males and females over 18 years of age with an estimated glomerular filtration rate (eGFR) of less than 30 ml/min/1.73m2 according to the Chronic Kidney Disease Epidemiology Collaboration (CKD-EPI) formula [10], who attended follow-up for at least 3 months in internal medicine clinics at the three hospitals from January 1, 2009, to December 31, 2019. Patients on chronic dialysis, who received renal transplantation or had an eGFR less than 15 ml/min/1.73m2 before referral were excluded. Patients on chronic dialysis, who received renal transplantation or had an eGFR less than 15 ml/min/1.73m2 before referral were excluded.

Patient data within 10 years before attending clinical follow-up was collected from the Clinical Data Analysis and Reporting System (CDARS), the data management system of all public hospitals and clinics managed by the Hospital Authority (HA) in Hong Kong.

Four categories of data were collected as features for model training and validation: Demographic, Biochemical, Pharmacological, and ICD-10 code. Demographic data, including age and gender, were recorded. Biochemical tests, including haemoglobin, haematocrit, haemoglobin A1C (Hba1c), serum sodium, potassium, urea, creatinine, alanine transaminase (ALT), aspartate aminotransferase (AST), bilirubin, uric acid, calcium, phosphate, bicarbonate, high-density lipoprotein (HDL), low-density lipoprotein (LDL), hepatitis serology, auto-immune markers, urine protein-creatinine ratio, and 24-h urine total protein, will be collected. Categorical data, including gender, ICD-10 codes, hepatitis serology, and auto-immune markers, were encoded using the one-hot approach. Each drug used in patients was recorded as one category with the total daily dosage as values.

**Fig. 1:**
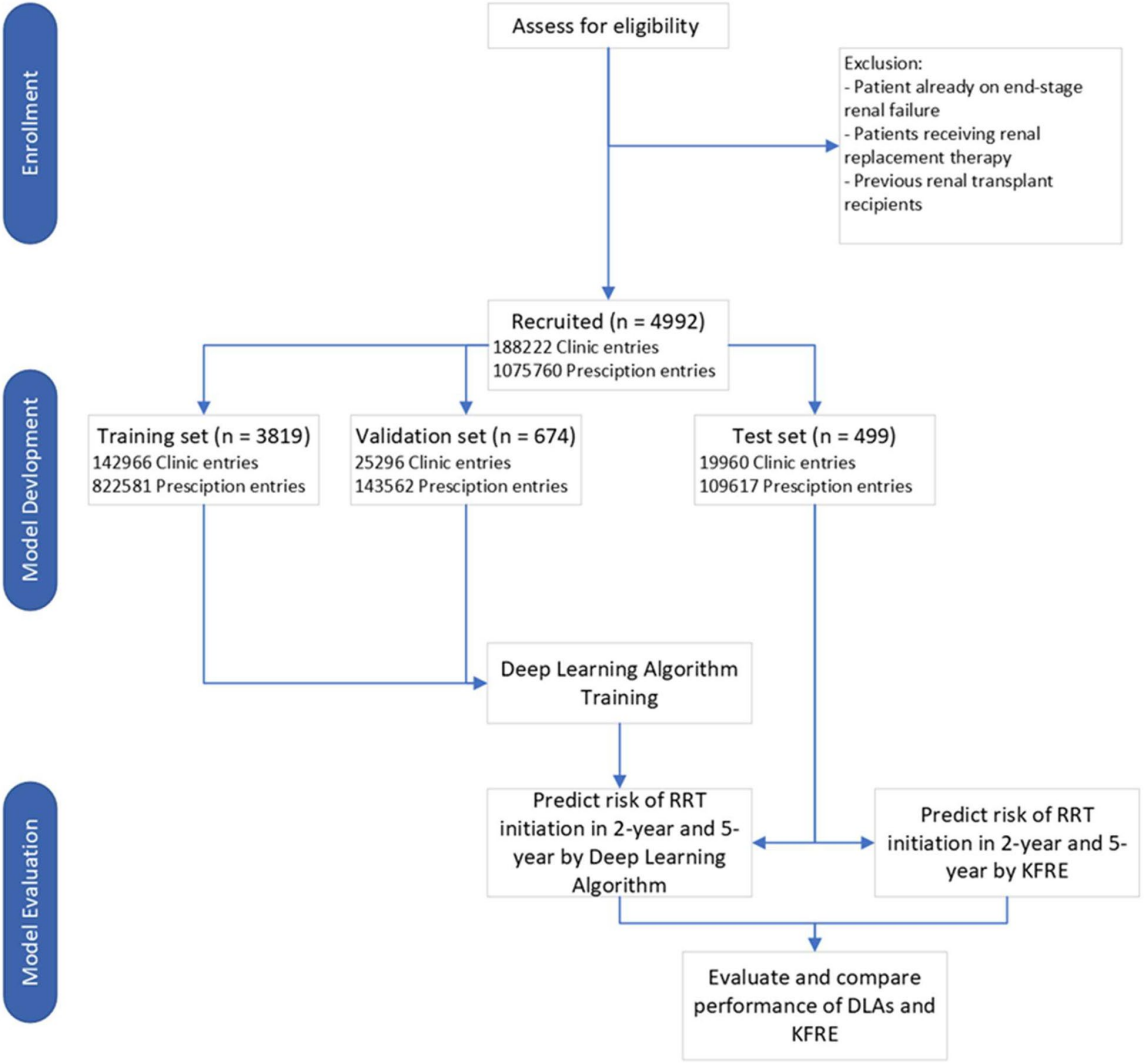
The flowchart of study population retrieval and splitting

To minimize data leakage, 10% of the patients were randomly selected and set aside as a test set. Their data would not be used in model training or validation. Fifteen per cent of the remaining training set was randomly selected and set aside as a validation set. The validation set was used during model training to prevent overfitting. The diagram of patient allocation this study is presented in Fig. 1.

Missing data for each clinical visit entry were imputed using the last observation carried forward (LOCF) approach [11]. The latest available value was used in model training for missing data. Unavailable prescription and biochemical investigation data were attributed with a value of 0 before feeding into the model as they were considered unprescribed or not done.

### Primary outcome

Data of all participants was collected till March 31, 2022. The primary outcome of this study was the initiation of renal replacement therapy (RRT). Each entry of clinical visit was labelled based on the date of starting RRT as one of the following: "no risk of RRT initiation in 5 years", "RRT initiated within 5 years", or "RRT initiated within 2 years". Data for each patient after initiation of RRT was discarded for analysis. This outcome was used to compare the model's performance with the Kidney Failure Risk Equation (KFRE).

### Explanatory data analysis

Pearson's correlation coefficient was used to assess the relationship between each continuous variable and the primary outcome. The correlation coefficient among the variables was computed to prevent multicollinearity. Before model training, all continuous variables were scaled to fit within 0 and 1.

We discarded all ICD-10 codes and drug prescriptions with frequencies less than 1% of the total training dataset entries to reduce overfitting and computational costs. Each variable underwent a Chi-square test with the primary outcome, and only variables with p-values less than 0.05 were included in the model training.

### Model training

Five supervised multi-classification DLAs were created and trained using TensorFlow 2.0 Python open-source machine learning platform [9], including convolutional neural network (CNN), artificial neural network (ANN), long short-term memory (LSTM), and two different combined networks base on the above 3 networks. The models were optimized using a randomized hyperparameter optimization algorithm provided by scikit-learn 1.1.3 Python open-source machine learning library [12]. The output of the model will be in the format of the probability of a patient belonging to each of the defined outcomes: "no risk of RRT initiation in 5 years," "RRT initiated within 5 years," or "RRT initiated within 2 years”.

### Model performance assessment

For each entry in the test set, the probabilistic categorization predictions of each DLA (CNN, ANN, LSTM, and their combinations) were compared with the ground truth to calculate their respective ROC-AUC and F1 scores. Since the dataset was imbalanced, these metrics were more appropriate than accuracy [13]. Sensitivity, specificity, PPV, and NPV were presented, too. All evaluations were computed using Python.

To make a fair comparison with the KFRE equation, each entry in the test dataset had its risk of RRT calculated by each KFRE Eq. [4]. Using a 50% probability as the cutoff, each clinical visit was classified into one of the following categories: no risk of RRT in 5 years, risk of RRT in 5 years, and risk of RRT in 2 years. The performance of the KFRE equation was then compared with the DLAs using a two-sided Mann–Whitney U test on 200 bootstrap samples per model.

## Results

### Patient characteristics

This study recruited a total of 4992 patients with 188,222 clinic visit entries. Data from 499 patients with 19,960 clinic visit entries were isolated as a test set and not used for model training to avoid data leakage and ensure unbiased performance evaluation. During the follow-up period, 1576 patients were diagnosed with Stage 5 CKD, and 989 patients required renal replacement therapy. The comparison of baseline characteristics between patients in the treatment and test sets is presented in Table 1. The comparison of patient baseline characteristics between the training and validation sets is presented in Table ST1 in the Supplementary documents.

**Table 1:** Comparison of baseline characteristics of patients in training/validation set and test set

Feature	Training and validation set patients *N* = 4493	Test set patients *N* = 499	*p*-value
G5 CKD progression during study period (%)	1437 (31.98%)	139 (27.86%)	0.067
RRT initiation during study period (%)	882 (19.63%)	107 (21.44%)	0.366
eGFR on recruitment (ml/min/1.73m2)	24.43 (24.32—24.56)	24.58 (24.24—24.93)	0.451
**Demographic data**			
Age (years) (95% CI)	70.54 (70.17—70.92)	70.72 (69.62—71.82)	0.771
Female gender (%)	1971 (43.87%)	216 (43.29%)	0.841
**Medical History**			
Hypertension	2585 (57.53%)	289 (57.92%)	0.908
Type 2 diabetes mellitus	1479 (32.92%)	172 (34.47%)	0.517
Heart failure	1035 (23.04%)	123 (24.65%)	0.182
Glomerular disorders	996 (22.17%)	107 (21.44%)	0.754
**Biochemical Data**			
Creatinine (µmol/L)	194.47 (192.97—195.98)	194.88 (190.1—199.66)	0.869
Urea (mmol/L)	13.07 (12.93—13.21)	12.97 (12.55—13.38)	0.628
ALT (U/L)	19.11 (18.64—19.58)	18.47 (17.39—19.56)	0.393
AST (U/L)	34.18 (29.32—39.04)	34.14 (24.27—44.01)	0.996
ALP (U/L)	87.40 (86.0—88.8)	88.22 (84.80 -s 91.65)	0.708
Albumin (g/L)	39.16 (39.02—39.3)	39.41 (39.01—39.81)	0.258
Urate (mmol/L)	0.47 (0.46—0.48)	0.47 (0.46—0.49)	0.627
Calcium (mmol/L)	2.32 (2.32—2.33)	2.34 (2.33—2.35)	0.019
Phosphate(mmol/L)	1.17 (1.17- 1.18)	1.19 (1.17—1.21)	0.071
Ca X PO4 (mg2/dL2)	33.75 (33.55—33.94)	34.56 (33.94—35.18)	0.011
Haemoglobin (g/L)	11.43 (11.4—11.8)	11.60 (11.4—11.8)	0.121
Haemoglobin a1c (%)	7.80 (7.73—7.87)	7.89 (7.7—8.09)	0.400
Spot urine protein: creatinine (mg/mg Cr)	2.20 (2.00—2.41)	1.75 (1.35—2.16)	0.156

The study identified 1100 unique ICD-10 codes from the clinical visit entries. ICD-10 Codes with less than 1060 counts, representing less than 1% of the total follow-up entries, were discarded, remaining 116 codes for chi-square testing. Among these, 95 ICD-10 codes were significantly correlated with the primary outcome (*p* < 0.05), as shown in Figure SF1 of the Supplementary document.

From 1,552,856 laboratory investigation results, 26 continuous and 14 categorical features were extracted. Two features, calcium-phosphate product and 24-h urine protein, were removed to prevent collinearity, while autoantibodies, except for anti-nuclear antibody (ANA), were also excluded due to high rates of missing data. The remaining 28 biochemical features were used for model training, and their distribution, Pearson correlation, and chi-square test results are presented in Figures SF2, SF3, and SF4 of the Supplementary document.

167 drugs were identified from 1,076,750 prescriptions in the training dataset. Using a student's t-test, 99 medications were statistically significant and selected for model training. The results of the t-test can be found in Table ST2 of the Supplementary document. All chosen features for model training are listed in Table ST3 of the supplementary document.

### Model training and optimization

Five different neural networks were developed in this study during model training and optimization. These networks included CNN, ANN, and LSTM, as well as two networks that combined these different layers differently. To optimize the performance of these networks, all of their parameters were optimized using a randomized hyperparameter optimization algorithm [10]. The details of the optimized hyperparameters and the structures of the neural network layers used in each model are included in Figures SF5-SF9 in the Supplementary document.

### Model evaluation

**Fig. 2:**
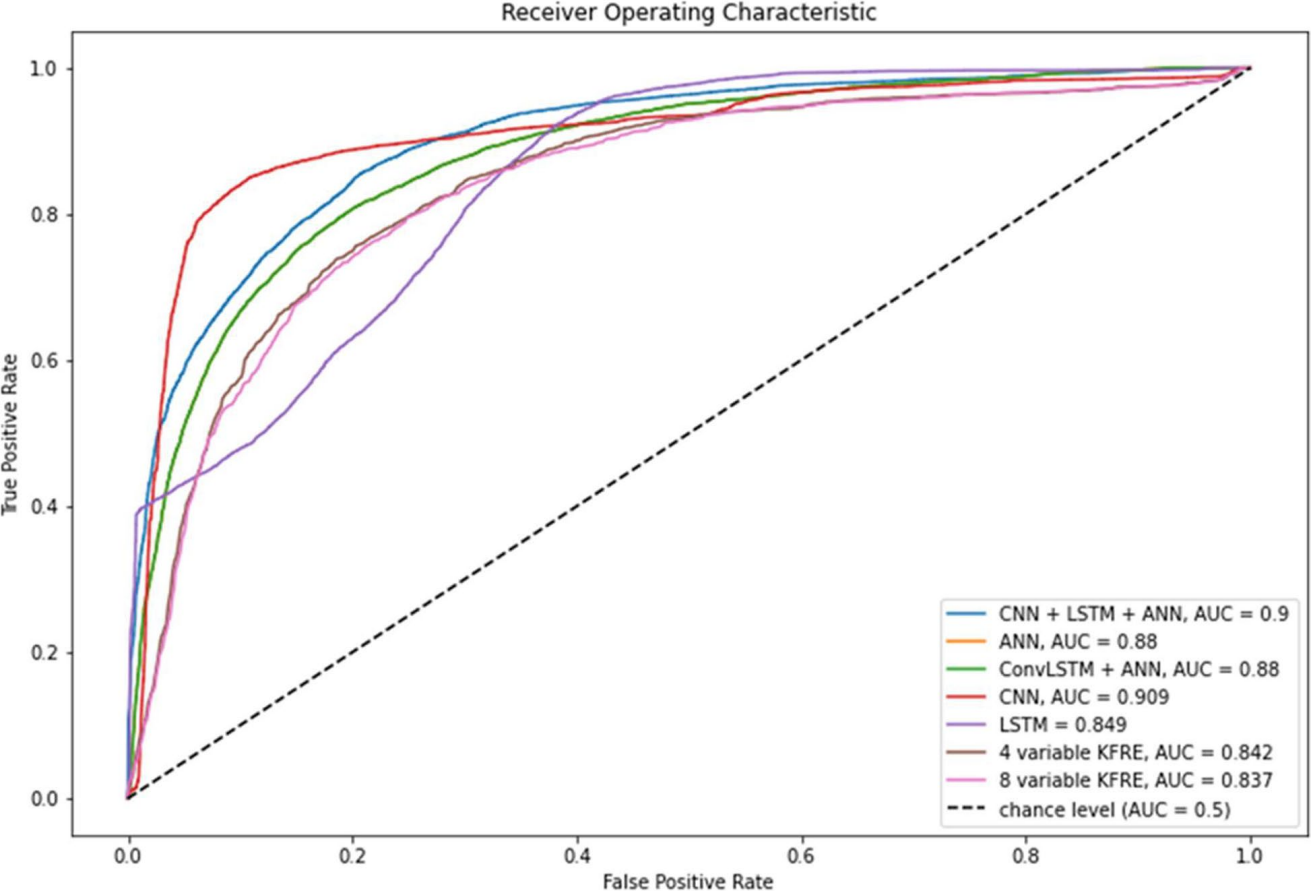
ROC curve of different deep learning models, using KFRE as comparison

**Fig. 3:**
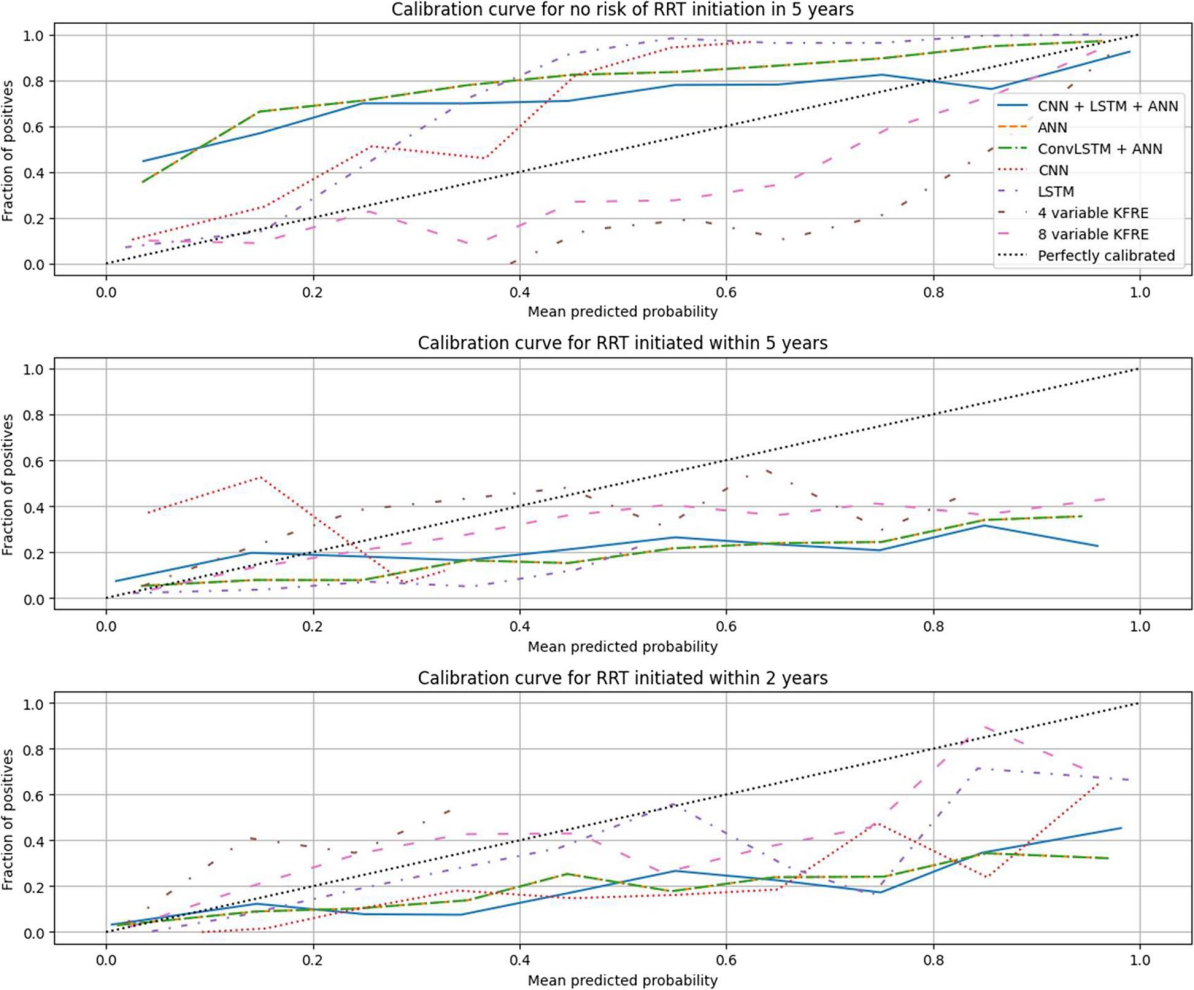
Calibration curve of different deep learning models and KFRE

All DLAs outperformed KFRE in predicting the risk of initiating RRT in 2 years and 5 years with significantly higher ROC-AUC scores (*p* < 0.001). The CNN algorithm showed the best overall performance with a ROC-AUC score of 0.91 (95% CI 0.907—0.914), F1 score of 0.79 (95% CI 0.781—0.795), specificity of 0.97 (95% CI 0.973—0.975), and PPV of 0.9 (95% CI 0.895—0.906). The 4-factor and 8-factor KFRE performed the worst with the lowest ROC-AUC score and sensitivity (Table 2), when using a threshold of 50%. Figure 2 presents the ROC curves of all DLAs and KFRE. Figure 3 presents the calibration curve for KFRE and DLAs.

**Table 2:** Evaluation of different deep learning models, using KFRE as comparison

	*ROC AUC*	*F1 score*	*Sensitivity*	*Specificity*	*PPV*	*NPV*
*CNN*	0.91 (0.907—0.914)	0.79 (0.781—0.795)	0.47 (0.465—0.474)	0.97 (0.973—0.975)	0.90 (0.895—0.906)	0.79 (0.784—0.787)
*CNN* + *LSTM* + *ANN*	0.90 (0.896—0.902)	0.76 (0.758—0.769)	0.74 (0.733—0.744)	0.88 (0.879—0.885)	0.76 (0.752—0.764)	0.87 (0.868—0.874)
*ANN*	0.88 (0.876—0.882)	0.76 (0.756—0.767)	0.71 (0.706—0.719)	0.87 (0.870—0.876)	0.74 (0.732—0.743)	0.86 (0.856—0.862)
*ConvLSTM* + *ANN*	0.88 (0.875—0.881)	0.76 (0.755—0.763)	0.71 (0.707—0.715)	0.87 (0.870—0.874)	0.73 (0.732—0.740)	0.86 (0.856—0.860)
*LSTM*	0.85 (0.844—0.852)	0.68 (0.673—0.684)	0.50 (0.493—0.505)	0.88 (0.875—0.881)	0.67 (0.664—0.679)	0.78 (0.776—0.781)
*KFRE (4 variable)*	0.84 (0.841—0.842)	0.32 (0.313—0.319)	0.40 (0.395—0.405)	0.75 (0.747—0.754)	0.42 (0.410—0.429)	0.88 (0.870—0.882)
*KFRE (8 variable)*	0.84 (0.836—0.837)	0.40 (0.394—0.406)	0.40 (0.395—0.405)	0.75 (0.747—0.754)	0.42 (0.410—0.429)	0.88 (0.870—0.882)

### Mislabeling analyze

To analyze mislabeling, we reviewed the ICD codes and prescriptions of patients mislabeled by the two most robust models (CNN and CNN + LSTM + ANN). While the CNN had fewer false positive predictions, the combined model demonstrated a better ability to identify patients who required dialysis after 5 years and those who underwent renal transplantation outside the local healthcare system. Among the false negative predictions made by both models, the combined model identified a higher proportion of patients with a risk of RRT (47 out of 80 patients, or 58.75%) and predicted RRT within 5 years for more than one-fourth of them (22 out of 80 patients, or 27.50%). Additionally, the combined model made fewer completely missed mislabeling (predicting that a patient had no risk of starting RRT while they initiated RRT in 5 years). Further details of the mislabeling analysis are provided in Table ST4 of the Supplementary document.

## Discussion

This study aims to find the most effective algorithm to predict the risk of starting RRT among CKD patients within a given period. This was done by DLAs and comparing their performance to that of the KFRE. To the best of the authors' knowledge, this was the first head-to-head comparison between KFRE and different neural networks using datasets of patients who had not been recruited in other studies before.

The study used different techniques in deep learning and developed five different models to predict the risk of starting RRT in two years and five years. All models were validated using a subset of patient data which was completely isolated from the dataset since the start of model development.

Of the five DLAs developed, the convolutional neural network (CNN) and a neural network combining CNN, long short-term memory (LSTM), and artificial neural network (ANN) layers showed the most accurate performance with the highest ROC-AUC score. In fact, the CNN model slightly outperformed the combined neural networks. This was unexpected as a more complex neural network was assumed to be able to consider more features and temporal relationships. The most likely reason for this was the relatively small training and validation dataset size causing overfitting in complex models. Further model development with larger datasets involving multiple centers should improve models' performance.

The review of the mislabeled patients, particularly false positives, proved that DLAs can pick up non-linear relationships among different features and provide predictions which may outperform the initial labelling system (Supplementary Table ST4). The less completely missed predictions and predicting uncoded renal transplantation of the combined model also suggested that a combined model may outperform single structured neural networks with adequate training data.

The superior performance of DLAs in this study has three implications. Firstly, a patient's medical history and recent prescriptions are essential features when estimating the risk of renal failure, and they should be included in any RRT risk estimation algorithms. Secondly, machine learning or deep learning can provide more accurate predictions than traditional linear, logistic, or Cox regression methods. Compared with the traditional methods, including temporal relationships during model development brings an advantage to neural networks. Thirdly, the potential to label unrecorded renal transplants and patients who started dialysis 5 years later has proven the ability of AI algorithms to recognize complex patterns that may not be apparent to humans.

This study offers a significant advantage by providing an enhanced, rapid, and automated prediction of RRT risk in CKD patients, eliminating the need for additional investigations and without disrupting existing protocols or workflows. The entire training and validation process can be conducted locally on a standard laptop computer. The implementation of AI-based DLAs can lead to better decision-making in clinical settings [14]. By accurately identifying patients who are more likely to require Renal Replacement Therapy (RRT), DLAs have the potential to reduce referrals that may not be necessary, particularly in cases where traditional methods might overestimate the risk of disease progression. The scalability of neural network training allows for efficient allocation of resources in local medical centers, optimizing patient care. In the era of growing CKD patient numbers and strained renal services, DLAs offer an objective and practical tool to assess RRT initiation risk, enabling patients to receive extended primary or community-based medical care before referral to specialist services.

To address the generalizability of these tools to primary care settings, where the scope of data collection might differ from that in specialized care. Our study leverages the extensive data available through the Hospital Authority, the sole public primary healthcare provider, which maintains a centralized patient record database ensuring comprehensive data collection and accessibility. This centralized system is instrumental in collecting demographic, biochemical, pharmacological, and ICD-10 code data, which are pivotal for the accuracy and applicability of our models. The interoperability between public and private healthcare sectors significantly enhances the utility of our models across diverse care settings. Private primary care providers have access to data recorded and maintained by public centers, thanks to established data-sharing protocols [15]. This interconnectivity ensures a broader data foundation, which is vital for the effective implementation of predictive models in primary care.

However, the study also has few limitations. Firstly, the neural networks developed in this study are still "black box" in nature, making it difficult for clinicians to explain and build rapport with patients when they need RRT. An explainable prediction by AI will also gain trust from clinicians to be more confident in applying them in clinical practice [16]. Implementing SHapley Additive exPlanations (SHAP) may help address this issue by showing important risk factors to patients in graphics [17].

Secondly, the performance of neural networks relies on the quality and quantity of training data. The data collected from the three medical centers predominantly represent the Chinese population, without recorded ethnicity. Implementing models trained solely on this data may introduce bias when applied to foreign medical centers. The usage of historical data from second tier clinics may introduce concept drift, which limiting the accuracy of the model when applying to a realistic primary care setting [18]. To handle missing data, our study utilized the Last Observation Carried Forward (LOCF) approach, suitable for our healthcare system's centralized data mechanism. In more decentralized systems, techniques such as Multiple Imputation using Chained Equations (MICE) or Probabilistic Principal Component Analysis (PPCA) should be considered [17].

Additionally, due to ethical constraints, the research team only had access to limited patient information, including ICD coding, prescriptions, and biochemical investigation reports, without consultation records. This may lead to imperfect patient labeling and model training, potentially excluding individuals who received prescriptions, renal transplantations, or hemodialysis in other countries. The low donation and transplantation rate in Hong Kong also limited the number of training data involving renal transplantation, possibly causing bias [19]. Recruiting data from other localities for training would be the most effective solution.

Thirdly, the study was limited by the hardware available, and the neural networks could only be optimized by a randomized hyperparameter optimization algorithm with 30 iterations. Other optimization methods, such as Bayesian algorithms, may produce better models but also consume more computation resources [20].

Lastly, while the data foundation is robust in terms of accessibility and interoperability, challenges remain, particularly in accessing and integrating private primary care data into public health systems. The variation in medication availability between public and private sectors, with public clinics offering a more limited selection within certain medication families, exemplifies the complexities of data integration across different healthcare settings. Addressing this issue is critical for the seamless application of AI-based predictive models in primary care, ensuring that patients across the healthcare continuum benefit from advanced, data-driven care methodologies.

Regarding the integration of our model into existing healthcare systems and handling the concept drift, we emphasize the necessity of specialized knowledge in Machine Learning Operations (MLOps) for managing data and automating processes. We believe that the design of data collection, management, and handling strategies should be a collaborative effort involving clinicians, data scientists, and machine learning experts prior to implementation. The balance between sensitivity and specificity is critical, and determining an appropriate threshold for clinical action is not solely a data science issue; it involves considering the tolerability of the local healthcare system, including manpower and budget constraints. This underscores the necessity for a collaborative approach between clinicians and data scientists to determine thresholds that optimize clinical utility without compromising patient safety. Currently, we are in the process of planning a prospective observational study to further validate the algorithm's performance in clinical settings. This step is crucial for ensuring that our model not only demonstrates theoretical efficacy but also practical applicability and integration into the existing healthcare infrastructure.

In our study, we trained our algorithm as a classification model. Our intention is to facilitate a clear and intuitive evaluation of our model's ability to predict high-risk patients requiring Renal Replacement Therapy (RRT). However, we acknowledge that this simplification may not entirely align with the continuous risk assessment provided by the KFRE and provide a suboptimal comparison. Presenting results as a median time or probability to RRT initiation is possible and may offer additional insights. Consequently, we suggest that future research could explore the development of a regression model using the same dataset, which might provide a different perspective on patient risk stratification.

Overall, the study's findings suggest that DLAs can be a valuable tool in predicting the risk of RRT in CKD patients. The ability of DLAs to identify complex patterns and non-linear relationships among different features can outperform traditional methods, such as linear, logistic or Cox regression models. The study also highlights the importance of including a patient's medical history and recent prescriptions as key features in risk estimation algorithms.

One potential application of these findings is the development of decision-support tools for clinicians. With the accurate predictions provided by DLAs, clinicians could use these tools to inform their clinical decision-making and improve patient care. For example, a tool that predicts the risk of RRT could help a clinician decide whether to refer a patient to a specialist, initiate specific treatments or implement lifestyle changes. Nevertheless, details of data pipeline design, storage, missing data handling and result interpretation should be openly discussed. Cooperation between data scientists, AI researchers and clinical care collegues are essential to implement AI in modern healthcare.

## Conclusion

In conclusion, this study demonstrated that deep learning models, particularly CNN and a combination of CNN, LSTM, and ANN layers, outperformed KFRE in predicting the risk of initiating RRT in CKD patients. The use of deep learning algorithms in predicting RRT risk provides a promising approach to minimizing manpower-related errors, reducing administration costs, and decreasing non-indicated referrals to speciality outpatient clinics, ultimately leading to better patient outcomes. Future research should focus on developing more optimized models with larger amounts of training data from different localities and developing methods to explain the decision-making process of deep learning algorithms to patients and clinicians.

### Supplementary Information

**Supplementary Material 1.**

## Data Availability

The data underlying this article will be shared on reasonable request to the corresponding author.

## Abbreviations

AKI: Acute kidney injury
ALT: Alanine transaminase
ANN: Artificial neural network
AST: Aspartate aminotransferase
CDARS: Clinical Data Analysis and Reporting System
CKD: Chronic kidney disease
CKD-EPI: Chronic Kidney Disease Epidemiology Collaboration
CNN: Convolutional neural network
DLAs: Deep learning algorithms
eGFR: Estimated glomerular filtration rate
ESRF: End-stage renal failure
HA: Hospital Authority
HDL: High-density lipoprotein
ICD-10: International Classification of Diseases, Tenth Revision
KFRE: Kidney Failure Risk Equation
LDL: Low-density lipoprotein
LOCF: Last observation carried forward
LSTM: Long short-term memory
NPV: Negative predictive value
POH: Pok Oi Hospital
PPV: Positive predictive value
RRT: Renal replacement therapy
ROC-AUC: Receiver operating characteristic—Area under the curve
SHAP: SHapley Additive exPlanations
TMH: Tuen Mun Hospital
TSH: Tin Shui Wai Hospital
